# 1594. Outcomes in Patients Receiving Long-Acting Cabotegravir-Rilpivirine in a Community, Infusion Center-Based Administration Model in Columbus, Ohio

**DOI:** 10.1093/ofid/ofad500.1429

**Published:** 2023-11-27

**Authors:** Natalie Nielsen, Bradley Taranto, Mohammad Mahdee Sobhanie, Ashley Lipps, Katherine Lehman, Susan L Koletar, Carlos Malvestutto, Yesha Patel

**Affiliations:** The Ohio State University Wexner Medical Center, Columbus, Ohio; The Ohio State University Wexner Medical Center, Columbus, Ohio; The Ohio State University, Columbua, Ohio; The Ohio State University Wexner Medical Center, Columbus, Ohio; The Ohio State University Wexner Medical Center, Columbus, Ohio; Ohio State University, Columbus, Ohio; The Ohio State University Wexner Medical Center, Columbus, Ohio; The Ohio State University Wexner Medical Center, Columbus, Ohio

## Abstract

**Background:**

Long-acting cabotegravir and rilpivirine (CAB/RPV) offers a promising alternative to daily oral antiretroviral therapy (ART); however, system-level and individual challenges in wide scale implementation are anticipated. We describe a community, infusion center-based model (ICBM) for administration of CAB/RPV and associated clinical outcomes.

**Methods:**

This was a single-center, retrospective cohort study of adults with HIV who were referred for enrollment in the ICBM (Figure 1) from 3/1/22 to 2/28/23 (Figure 2). We investigated demographics, system-level implementation variables, individual factors, and clinical outcomes among CAB/RPV recipients.

**Figure 1:**
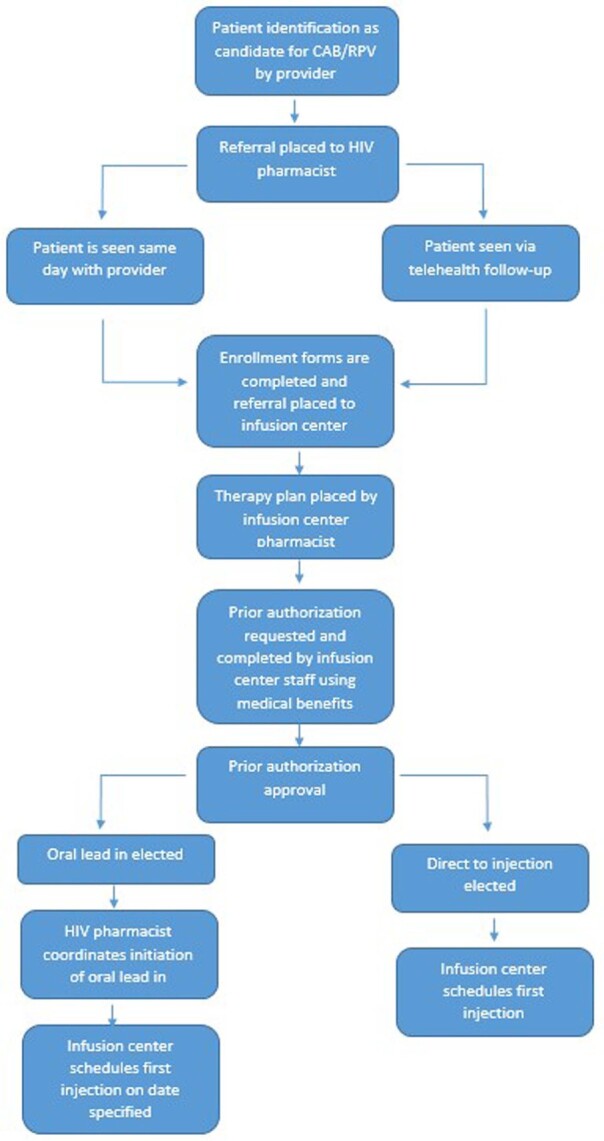
Community, Infusion Center-Based Model

**Figure 2:**
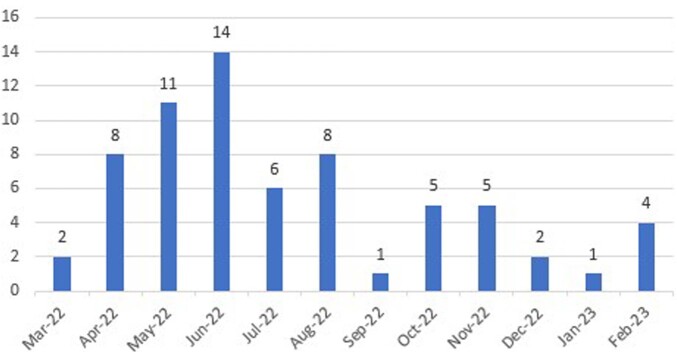
Number of Enrollments per Month in the Infusion Center-Based Model

**Results:**

79 patients were referred for enrollment in the ICBM and 64 patients received at least 1 dose of CAB/RPV. Reasons for CAB/RPV not being administered include insurance barrier, N=2 (13.3%); changed mind, N=3 (20%); unable to reach, N=2 (13.3%), difficulty with collecting labs, N=1 (6.7%), and CAB/RPV started outside of study period, N=4 (26.7%). Of those who received at least one dose of CAB/RPV, 79.9% were male, 48.4% were African American, 42.4% were Medicaid beneficiaries, 67.2% reported mental illness and 39.1% reported alcohol or non-tobacco substance use. Other baseline factors are listed in Table 1. Mean time from referral to first injection was 38.8 days excluding oral lead in time. Mean treatment duration was 176 days (range 20-326 days). 211 maintenance injections were administered during the study period; 16 (7.6%) were outside of the injection window with 10 (62.5%) oral bridges administered. All patients were virally suppressed at end of study period. Overall, 1,122 interventions were completed [HIV pharmacist, N=327 (29.1%); clinic staff, N=26 (2.3%); infusion center pharmacist, N=364 (32.4%); and infusion center staff, N=405(36.1%)]. Table 2 describes interventions performed.

**Table 1:**
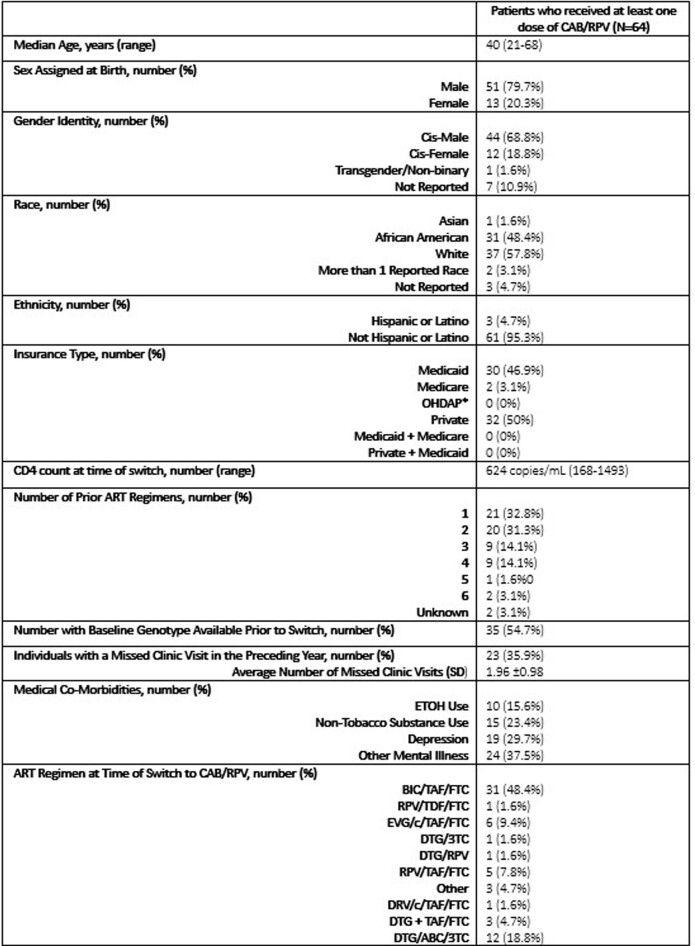
Baseline Demographics & Clinical Factors. †Abbreviations: OHDAP, Ohio HIV Drug Assistance Program; ART, antiretroviral therapy; PI, protease inhibitor; NNRTI, non-nucleoside reverse transcriptase inhibitor; INSTI, integrase strand transfer inhibitor; NRTI, nucleoside/nucleotide reverse transcriptase inhibitor; ATV/r, atazanavir/ritonavir; DRV/r, darunavir/ritonavir; DRV/cobicistat, darunavir/cobicistat; RPV, rilpivirine; EFV, efavirenz; RAL, raltegravir; EVG/cobicistat, elvitegravir/cobicistat; DTG, dolutegravir; BIC, bictegravir; TDF/FTC, tenofovir disoproxil fumarate/emtricitabine; TAF/FTC, tenofovir alafenamide/emtricitabine; ABC/3TC, abacavir/lamivudine. *LA CAB/RPV is not covered under formulary for OHDAP

**Table 2:**
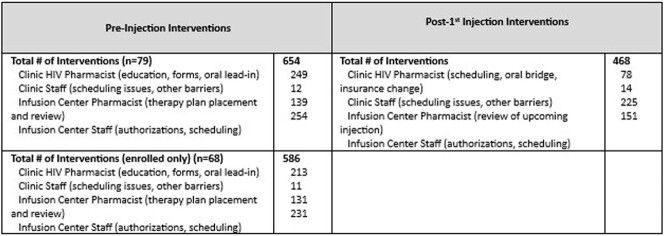
Collaborative Interventions Completed within Infusion Center-Based Model

**Table 3.**
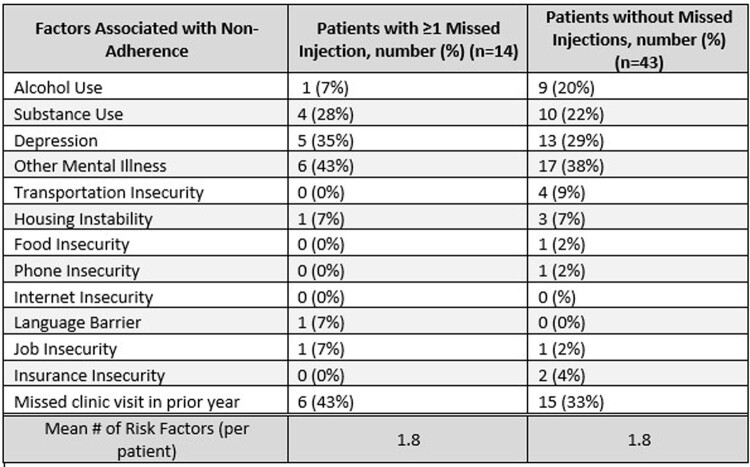
Adherence Barriers Among Those on Long-Acting Cabotegravir-Rilpivirine

**Conclusion:**

Implementation of CAB/RPV requires a collaborative effort to address system-level and individual challenges. Utilizing existing infrastructure allows for resource optimization to engage vulnerable populations and enhance equitable access. Our program shows successful treatment with CAB/RPV in individuals with adherence barriers (Table 3) at community infusion centers.

**Disclosures:**

**Carlos Malvestutto, MD MPH**, Pfizer: Advisor/Consultant|ViiV Healthcare: Advisor/Consultant

